# Rapid automated quantification of left ventricular ejection fraction with LV-METRIC – a novel segmentation algorithm

**DOI:** 10.1186/1532-429X-11-S1-P117

**Published:** 2009-01-28

**Authors:** Noel CF Codella, Matthew D Cham, Richard Wong, Christopher Chu, Kirsten Healy, Martin R Prince, Yi Wang, Jonathan W Weinsaft

**Affiliations:** Weill Medical College of Cornell University, New York, NY USA

**Keywords:** Stroke Volume, Cardiac Magnetic Resonance, Segmentation Algorithm, Automate Segmentation, Phase Contrast Imaging

## Objective

To evaluate diagnostic performance and time saved by LV-METRIC, a novel automated algorithm for LV quantification, among a broad unselected clinical patient population undergoing CMR.

## Background

Cardiac magnetic resonance (CMR) is an imaging standard for quantification of LV ejection fraction (EF) and volume. CMR typically relies on manual tracing (MT). MT can be time consuming and operator-dependent. Automated segmentation holds potential for rapid LV quantification. Established automated algorithms often fail or require extensive user interface, possibly due to the fact that they employ assumptions regarding cavity shape or regional propagation. We developed an automated segmentation algorithm (LV-METRIC) that involves no geometric assumptions and instead quantifies LV EF and volume based on local per-pixel signal intensity while accounting for partial voxel effects. The purpose of the current study was three-fold; (1) to evaluate LV-METRIC performance vs. MT among a broad unselected patient population; (2) to compare processing time by LV-METRIC to MT, and (3) to compare LV-METRIC and MT to an independent standard of LV flow quantification.

## Methods

LV-METRIC and MT were independently applied for quantification of LV EF and volumes on consecutive patients that underwent CMR (1.5 T) between June-November, 2007. Processing times were recorded. Quantification was based on endocardial border delineation of consecutive short axis SSFP images throughout the LV (slice thickness 6 mm, gap 4 mm, typical spatial resolution 1.9 × 1.4 mm). Through-plane aortic valve phase contrast imaging was performed on a subset (n = 65) of patients for independent confirmation of LV stroke volume. No patients were excluded based on clinical characteristics; the only exclusion criteria were cardiac arrhythmias or image artifacts that prohibited MT (6.7% patients).

## Results

**Figure 1:**
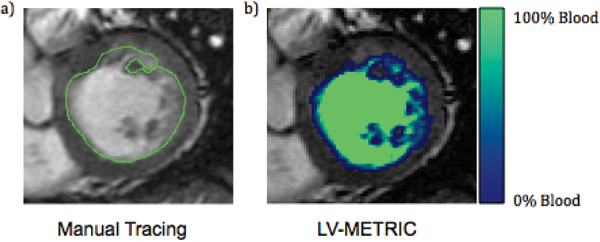
Figure 1

The study population was comprised of 151 patients (54 ± 14 yo, HTN 40%, DM 23%, CAD 34%). LV-METRIC was successful in all patients; average processing time was 22 seconds for LV-METRIC and 4 minutes 59 seconds for MT (p < 0.001). An example segmentation is shown in Figure 1, where MT is depicted in Fig. 1a, and LV-METRIC in Fig. 1b. Voxel blood content color labels for LV-METRIC are also illustrated. LV EF based on LV-METRIC was, on average, within 2 percentage points of MT irrespective of whether partial voxel (-2.0 ± 2.3%) or full voxel (0.6 ± 2.3%) computation was used (Table 1). LV volumes yielded by LV-METRIC were smaller than MT. Mean end-diastolic and end-systolic volumes in LV-METRIC measurements with partial voxel interpolation were 25.7 mL and 14.3 mL smaller than MT measurements, respectively. Full voxel computation yielded less drastic differences, with diastolic volumes smaller by 4 mL and systolic volumes larger by 1.4 mL. All EF and volumetric differences between LV-METRIC and MT were significant (p < 0.001). Correlations for cavity volumes, global stroke volume, and ejection fraction were high (all R^2^ > .95). Both LV-METRIC and MT similarly agreed with phase contrast (all differences p < 0.05); Although volumetric stroke volume by MT was, on average, similar to PC (Δ5.9 mL), there was substantial variance between the two techniques (SD ± 13.4 mL). Similar differences were evident when comparing PC and LV-METRIC with (6.2 ± 14.2 ml) or without (-3.9 ± 14.7 ml) partial voxel interpolation.

**Table 1:** Comparison of LV-METRIC to manual tracing

	Manual tracing	LV-METRIC (PV interpolation)	Δ	LV-METRIC (no PV interpolation)	Δ
Ejection fraction	57.1 ± 17.0	59.1 ± 17.4	-2.0 ± 2.3	56.5 ± 16.7	0.6 ± 2.3
End-diastolic volume (ml)	157.8 ± 67.9	132.1 ± 60.5	25.7 ± 10.9	153.8 ± 65.9	4.0 ± 6.8
End-systolic volume (ml)	75.1 ± 62.7	60.9 ± 55	14.3 ± 9.6	73.7 ± 60	1.4 ± 5.5
Stroke volume (ml)	82.6 ± 26.8	71.2 ± 24.1	11.4 ± 6.6	80 ± 26.9	2.6 ± 5.3
Processing time (minutes)	5:18 ± 1:56	0:22 ± 0:13	4:37 ± 1:51

## Conclusion

Among a broad population of consecutive patients, LV-METRIC, a novel automated CMR segmentation algorithm, provides highly accurate quantification of LV ejection fraction and volumes with a marked reduction in processing time vs. MT. Both LV-METRIC and MT provide similar agreement with an independent standard of phase contrast imaging.

